# Serum Selenium and Ceruloplasmin in Nigerians with Peripartum Cardiomyopathy

**DOI:** 10.3390/ijms16047644

**Published:** 2015-04-07

**Authors:** Kamilu M. Karaye, Isah A. Yahaya, Krister Lindmark, Michael Y. Henein

**Affiliations:** 1 Department of Medicine, Bayero University and Aminu Kano Teaching Hospital, PO Box 4445, Kano, Nigeria; 2 Department of Public Health and Clinical Medicine, Umea University, SE-901 87 Umea, Sweden; E-Mails: krister.lindmark@vll.se (K.L.); michael.henein@umu.se (M.Y.H.); 3 Department of Chemical Pathology, Bayero University and Aminu Kano Teaching Hospital, Kano, Nigeria; E-Mail: yahadagiri2@yahoo.com; 4 Department of Cardiology, Umea Heart Centre, SE-901 85 Umea, Sweden

**Keywords:** peripartum cardiomyopathy, risk factors, selenium deficiency, ceruloplasmin, heart failure

## Abstract

The study aimed to determine if selenium deficiency, serum ceruloplasmin and traditional birth practices are risk factors for peripartum cardiomyopathy (PPCM), in Kano, Nigeria. This is a case-control study carried out in three hospitals, and PPCM patients were followed up for six months. Critically low serum selenium concentration was defined as <70 µg/L. A total of 39 PPCM patients and 50 controls were consecutively recruited after satisfying the inclusion criteria. Mean serum selenium in patients (61.7 ± 14.9 µg/L) was significantly lower than in controls (118.4 ± 45.6 µg/L) (*p <* 0.001). The prevalence of serum selenium <70 µg/L was significantly higher among patients (76.9%) than controls (22.0%) (*p <* 0.001). The mean ceruloplasmin and prevalence of socio-economic indices, multiparity, pregnancy-induced hypertension, obesity and twin pregnancy were not different between the groups (*p >* 0.05). Logistic regression showed that rural residency significantly increased the odds for serum selenium <70 µg/L by 2.773-fold (*p =* 0.037). Baseline serum levels of selenium and ceruloplasmin were not associated with six-month mortality. This study has shown that selenium deficiency is a risk factor for PPCM in Kano, Nigeria, and is related to rural residency. However, serum ceruloplasmin, customary birth practices and some other characteristics were not associated with PPCM in the study area.

## 1. Introduction

Peripartum cardiomyopathy (PPCM) is one of the most common causes of heart failure (HF) among women in Kano, other parts of northern Nigeria and Africa [1,2]. Its etiology and pathogenesis is still unknown, although several hypotheses have been proposed over the years, including selenium deficiency [3,4].

The Hausa-Fulani tribe of northern Nigeria was described decades ago to have the highest known incidence of peripartum cardiac failure (PPCF) which was linked to some peculiar local puerperal customs which were practiced for as long as 3 months from the time of delivery [5,6,7]. These include twice-daily hot baths, of which the leaves of local trees were used to splash very hot water on the body for about 15–30 min, “wankan jego” (in Hausa language), lying on beds made of clay which is heated from below with firewood, in spite of the hot weather, and frequent ingestion of pap made from millet and enriched with dried lake salt, “Kunun Kanwa” (in Hausa language) [5,6,7]. The practices are believed to stimulate/enrich breast milk in the immediate postpartum period and improve the overall health of the mother.

Epidemiologic studies showed that low selenium levels in the soil and in local foodstuffs correlated with low selenium levels in whole-blood and hair samples, and treatment with sodium selenite prevented Keshan disease and mitigated the clinical manifestations in patients with the disease [8,9,10,11,12]. In 1992, Cenac *et al.* reported that selenium deficiency might be an important problem in Sahelian African patients with PPCM [4]. In contrast, Fett *et al.* reported low selenium levels (<70 ng/mL) in only one out of 18 PPCM patients in Haiti [13].

Ceruloplasmin is a blue colored plasma protein that binds up to 95% of circulating copper [14]. Ceruloplasmin has recently been shown to be raised in patients with ischemic and non-ischemic cardiomyopathy as an acute phase reaction to HF, and to correlate positively with C-reactive protein and inversely with left ventricular ejection fraction (LVEF) [15]. To the best of our knowledge, the relationship between ceruloplasmin and PPCM has not been previously studied.

The present study therefore aimed to assess the significance of serum levels of selenium and ceruloplasmin as well as the previously described traditional birth practices, as PPCM risk factors, in Kano, Nigeria.

## 2. Results and Discussion

### 2.1. Results

A total of 54 PPCM patients and 77 controls were consecutively screened, but only 39 patients and 50 controls were recruited after satisfying all the inclusion criteria, including availability of serum selenium and ceruloplasmin results. The baseline characteristics are presented in Table 1. The mean age, systolic and diastolic blood pressures, body mass index, monthly family income and years of education were not different (*p >* 0.05) between the two groups, but PPCM patients had significantly higher heart rates (*p* < 0.001). Screening for human immunodeficiency virus (HIV) was not carried out, and none of the subjects gave such history. All the subjects were in sinus rhythm, but premature ventricular or atrial extrasystoles were seen in three (7.7%) patients and two (4.0%) controls (*p =* 0.453; not significant). The mean LVEF among patients was 33.4% ± 10.1%, and two (5.1%) patients had LVEF of 45% to 49%. The median time from childbirth to presentation was 7.0 (range = 1–39) weeks among patients and 6.0 (range 1–31) weeks among controls (*p =* 0.589). However, some symptoms of HF started during the *puerperium* among all patients. All blood samples were taken at the time of evaluation and recruitment.

**Table 1:** Baseline characteristics of peripartum cardiomyopathy (PPCM) patients and controls.

Variables	PPCM Patients *n* = 39	Controls *n* = 50	*p*-Value
Age, years	25.9 ± 6.5	26.4 ± 6.2	0.674
Body mass index, kg/m^2^	21.2 ± 3.9	21.9 ± 4.2	0.377
Systolic blood pressure, mmHg	119 ± 26	126 ± 18	0.124
Diastolic blood pressure, mmHg	86 ± 19	83 ± 14	0.469
Hypotension (SBP *<* 100 mmHg)	13 (33.3%)	2 (4%)	<0.001 *
Heart rate, beats/min	108 ± 17	94 ± 16	<0.001 *
Log_10_ Family income/month, ₦	4.31 ± 0.31	4.28 ± 0.37	0.672
Log_10_ Education, years	0.99 ± 0.21	0.97 ± 0.15	593
NYHA class			
I	0	50 (100%)	<0.001 *
II	14 (35.9%)	0	
III	18 (46.2%)	0	
IV	7 (18.0%)	0	
Hemoglobin, g/dL	12.3 ± 1.8	13.4 ± 2.8	0.058
Serum sodium, mmol/L	136.0 ± 5.5	140.4 ± 4.1	<0.001 *
Serum creatinine, µmol/L	79.8 ± 21.9	73.3 ± 18.0	0.149
Anemia	16 (41.0%)	11 (22.0%)	0.053
Serum albumin, g/dL	32.4 ± 4.9	35.8 ± 7.3	0.061
Log_10_ ALT	1.17 ± 0.47	1.27 ± 0.29	0.305
Log_10_ AST	1.29 ± 0.40	1.43 ± 0.23	0.088
ACEI or ARB	18 (46.2%)	0	-
Digoxin	37 (94.9%)	0	-
Beta-blockers	3 (7.7%)	0	-
Spironolactone	37 (94.9%)	0	-
Nifedipine	0	7 (14.0%)	-
α-Methyl Dopa	5 (12.8%)	14 (28.0%)	0.083
Thiazide diuretic	10 (25.6%)	8 (16.0%)	0.261

₦, Nigerian Naira; * *p*-value statistically significant; SBP, systolic blood pressure; NYHA, New York Heart Association; ALT, alanine transaminase; AST, aspartate transaminase; ACEI, Angiotensin Converting Enzyme Inhibitors; ARB, Angiotensin Receptor Blocker. Results are presented as means ± standard deviations, or as numbers with percentages in parentheses.

Risk factors for PPCM are compared in Table 2. The mean serum selenium for patients (61.7 ± 14.9 µg/L) was significantly lower than for controls (118.4 ± 45.6 µg/L) (*p <* 0.001). The prevalence of serum selenium levels of <70 and <45 µg/L were also significantly higher among the patients than controls (*p <* 0.001 for both comparisons). The mean ceruloplasmin and prevalence of high ceruloplasmin, multiparity, pregnancy-induced hypertension, obesity and twin pregnancy were not significantly different between the two groups (*p >* 0.05). The prevalence of the traditional birth practices (traditional hot baths and use of “kunun kanwa”) were remarkably higher among the controls than PPCM patients (*p <* 0.001), while rural residency was significantly higher among the patients than controls (*p =* 0.001). None of the subjects had ever used or seen the clay beds.

**Table 2:** Profile of risk factors for peripartum cardiomyopathy among PPCM patients and controls.

Variables	PPCM Patients *n =* 39	Controls *n =* 50	*p*-Value
Selenium, µg/L	61.7 ± 14.9	118.4 ± 45.6	<0.001 *
Selenium <70 µg/L	30 (76.9%)	11 (22.0%)	<0.001 *
Selenium <45 µg/L	6 (15.4%)	0	-
Ceruloplasmin, mg/dL	43.9 ± 15.8	45.3 ± 15.7	0.682
Ceruloplasmin >60 mg/dL	7 (18.0%)	4 (8.0%)	0.201
Multiparity	29 (74.4%)	42 (84.0%)	0.296
Pregnancy-induced hypertension	16 (41.0%)	14 (28%)	0.197
Traditional hot baths	14 (35.9%)	41 (82%)	<0.001 *
“Kunun kanwa”	9 (23.1%)	39 (78%)	<0.001 *
Twin pregnancies	2 (5.1%)	2 (4.0%)	0.799
Rural residency	18 (46.2%)	7 (14.0%)	<0.001 *

* *p*-Value statistically significant; Results are presented as means ± standard deviations, or as numbers with percentages in parentheses.

The range of serum selenium was 28.2–100.3 µg/L in patients and 58.8–240.2 µg/L in controls. The 25th, 50th and 75th percentiles of serum selenium were 53.3, 61.5 and 69.7 µg/L among patients, and 82.6, 112.3 and 147.1 µg/L in controls, respectively. Further analysis using binary logistic regression showed that the odds for having serum selenium <70 µg/L was increased by rural residency by 2.773-fold (OR = 2.773; CI = 1.063–7.234; *p =* 0.037). In addition, serum selenium correlated positively (*r* = 0.236; *p =* 0.026) with years of education, but not with haemoglobin, age, LVEF, family income, parity, blood pressures, BMI or serum creatinine of the subjects (*p >* 0.05).

At six months follow up, a total of seventeen (43.6%) patients were evaluated, six (15.4%) had died and the remaining sixteen (41.0%) patients were lost to follow up. Although *postmortem* examination was not carried out on any of the deceased patients, information gathered from close relatives suggested that they all died of worsened HF. The baseline characteristics of the deceased including serum selenium were compared to those of survivors in Table 3, and showed no significant differences between the groups except in their monthly family income. The deceased had significantly lower Log_10_ income than survivors (4.1 ± 0.1 *vs.* 4.4 ± 0.3 respectively; *p =* 0.003). The monthly income was however not a predictor of mortality (*p =* 0.099) when assessed in a binary logistic regression model. Furthermore, the mean LVEF of the 17 survivors increased significantly from 33.3% ± 10.5% at baseline to 41.9% ± 12.7% at follow-up (*p =* 0.039), and three patients (17.7%) had LVEF ≥55% at six months.

**Table 3:** Baseline characteristics of patients alive and the deceased at six-month follow-up.

Variables	Patients Alive *n =* 17	Deceased *n =* 6	*p*-Value
Selenium, µg/L	63.3 ± 14.1	60.1 ± 13.5	0.634
Ceruloplasmin, mg/dL	49.1 ± 13.2	39.4 ± 10.8	0.127
Age, years	26.3 ± 5.9	26.0 ± 9.2	0.940
NYHA class			
II	7 (41.2%)	3 (50.0%)	0.280
III	8 (47.1%)	1 (16.7%)	
IV	2 (11.8%)	2 (33.3%)	
Systolic BP, mmHg	112.8 ± 19.2	120.0 ± 32.3	0.525
Diastolic BP, mmHg	79.8 ± 14.6	91.7 ± 24.0	0.169
Heart rate, beats/min	107 ± 19	114 ± 13	0.379
Log_10_ income	4.4 ± 0.3	4.1 ± 0.1	0.003 *
BMI, Kg/m^2^	21.1 ± 4.9	21.3 ± 2.4	0.933
Log_10_ creatinine	1.86 ± 0.16	1.90 ± 0.15	0.622
Hemoglobin, g/dL	12.9 ± 1.4	13.0 ± 1.0	0.929
LVEDDi, mm/m^2^	40.4 ± 5.4	41.4 ± 4.4	0.683
LVEF, %	33.3 ± 10.5	29.2 ± 12.8	0.446
ARB/ACEI	8	4	0.646
Beta blockers	1	1	0.481
Spironolactone	15	6	1.000
Digoxin	16	5	0.273

NYHA, New York Heart Association; BP, blood pressure; BMI, body mass index; LVEDDi, left ventricular end-diastolic dimension indexed to body surface area; LVEF, left ventricular ejection fraction; ARB, angiotensin II receptor blocker; ACEI, angiotensin converting enzyme inhibitor; * *p*-value statistically significant. Results are presented as means ± standard deviations, or as numbers with percentages in parentheses.

When the sixteen patients (41.0%) lost to follow-up were compared to the 17 survivors (43.6%), the former group were found to have significantly lower hemoglobin (11.6 ± 2.1 g/dL) than the latter (12.9 ± 1.4 g/dL) (*p =* 0.044). However, other differences between the groups were not statistically significant (*p >* 0.05).

### 2.2. Discussion

The present study has shown that low serum selenium and rural residency were highly significant risk factors for PPCM in Kano, north-western Nigeria. In addition, rural residency increased the odds for having critically low serum selenium levels by up to 2.77-fold. The study has also shown that ceruloplasmin, socio-economic indices, multiparity, pregnancy-induced hypertension, obesity and twin pregnancy were not risk factors for PPCM in the study area. Traditional birth practices that have previously been associated with PPCF in northern Nigeria were still being commonly practiced by most women recruited in the study but were not PPCM risk factors.

#### 2.2.1. Selenium Deficiency as PPCM Risk Factor

Selenium is a naturally occurring element found in soil, rocks and water [16]. The selenium content in food principally depends on the concentration and physico-chemical forms existing in the soil [16]. Although food is the main source of selenium for man, the dietary selenium intake principally depends on the region of origin of the foodstuffs and the protein content [16,17]. Thus, animal products (meat and fish products, especially shrimp) tend to be richer in selenium than plant materials [18]. It is also important to appreciate that levels of serum selenium are determined by many factors, including its availability in foods, absorption, cooking, lactation, alcohol, chronic illnesses, *etc.* [16,19]. Cenac *et al.* reported for the first time from Niger Republic, where PPCM is an endemic disease, that selenium deficiency may be an important problem in Sahelian African patients with PPCM, akin to what was described for Keshan disease [4,8,9,10,11,12]. In support of the PPCM selenium theory by Cenac *et al.*, our results have shown critically low selenium levels among 76.9% of the studied PPCM patients [1,4]. North-western Nigeria shares a long border, geography and customs with Niger Republic, hence the common food types and dietary habits, which are the sources of selenium. Our results have shown that PPCM patients have significantly lower serum selenium levels and significantly higher prevalence of rural residency than controls in spite of similar income and educational levels. In addition, rural residency significantly increased the odds of having critically low serum selenium levels. It is well known that in Nigeria, most rural residents are subsistence farmers who tend to consume the locally produced food and grown animals. Urban residents on the other hand are exposed to imported foods and animals, from regions where there is no selenium deficient soil and animals. Therefore, it is reasonable to hypothesize that most women in Kano (and the Sahel region) develop PPCM if they depend on locally produced foods and animals. The serum selenium levels among PPCM patients in Kano (61.7 ± 14.9 µg/L) and Niamey (48.0 ± 25 µg/L) are similar, most likely because of their geographical and cultural similarities, which explains the heavy burden of the disease in the region, in comparison with respective values in Haiti of 110 µg/L (range 67–145 µg/L) [4,13]. This difference could be explained on the basis of geographical and cultural factors. In spite of the foregoing, the question still remains: does selenium deficiency have a cause-and-effect relationship with PPCM? For a convincing answer, similar epidemiologic studies to those carried out in China are needed, where Keshan disease has been relegated to history [11].

In the present study, there was no relationship between baseline serum selenium or ceruloplasmin and mortality at 6 months follow-up. The mortality rate of 26.1% was higher than what was reported from South Africa (11.6%) at six months follow up [20], perhaps because of a high prevalence of hypotension, poverty and poor prescription patterns by the physicians among other factors that precluded adequate usage of guideline-recommended treatments such as angiotensin converting enzyme inhibitors and beta-blockers. Although the attrition rate was high (41%) as expected, patients lost to follow-up were in most respects similar to those who survived or died.

Selenium is a key component of the antioxidant glutathione peroxidase. A decrease in the activity of this enzyme secondary to selenium deficiency may lead to an increase in the oxidative stress in myocardial cells, and this can make the cells more susceptible to infections, especially by viruses [21]. This mechanism was demonstrated in mice models by injecting viral genomes into mice deficient in selenium, and compared with mice with good nutritional support. The incidence of myocarditis in the first group was much higher [21].

#### 2.2.2. PPCM and Traditional Birth Practices Peculiar to Northern Nigeria

About four decades ago, some studies described an association between puerperal customary practices and PPCF in Zaria, a city about 120Km from Kano in north-western Nigeria [5,6,7]. Davidson *et al.* reported that the customs of Hausa women in Zaria were important in the pathogenesis of PPCF; only 1% did not take postpartum baths, 3% did not lie on the hot beds, and 6% took no “kanwa” [7]. It is important to note that the Zaria syndrome of PPCF was defined as a “high-output HF with well preserved ventricular function”, making it different from what is known today as PPCM [3,6]. In a cross-sectional study (without a control group), Isezuo *et al.* reported that up to 81.5% of PPCM patients in Sokoto practiced the hot baths for 30 days [22]. In our case-control study, the hot baths and intake of “kunun kanwa” were still commonly practiced by the great majority of healthy women *postpartum*. The prevalence of both practices was significantly lower among the PPCM patients than among the healthy controls. Therefore, the notion that those traditional birth practices are PPCM risk factors should be downplayed.

#### 2.2.3. Other PPCM Risk Factors

Unlike previous reports, the present study has shown that socio-economic indices, multiparity, pregnancy-induced hypertension, obesity and twin pregnancy were not risk factors for PPCM in the study area [2,23]. In addition, the study has shown for the first time that CP was not associated with PPCM, in contrast to a recent report that found it to be related to some biochemical changes and LV systolic dysfunction in heart failure [15]. These findings have therefore proven that PPCM is a multi-factorial disease, and the relative importance of risk factors varies between individuals and geographic regions.

Selenium levels in the soil and domestic animals in Kano, as well as in other geographical regions are relevant information but beyond the scope of the present study.

#### 2.2.4. Limitations

Although relevant, blood levels of vitamins, C-reactive proteins, natriuretic peptides, thyroid hormones and prolactin were not assessed in the present study. Still the present study has shed more light on the selenium theory of PPCM and has discarded the theory of traditional birth practices as risk factors for PPCM. Future studies should ideally be community-based, prospective and comparative between various regions, and should involve assessment of selenium in the environment (foods and soil) as well as blood levels of the above-mentioned biomarkers.

## 3. Experimental Section

The study was carried out in Aminu Kano Teaching Hospital, Murtala Mohammed Specialists’ Hospital (MMSH) and Cardia Heart Clinic, Kano, Nigeria, in two phases. The initial case-control study phase was followed by a longitudinal arm in which only the patients were followed up for six months.

### 3.1. Clinical Evaluation

Before the commencement of the study, the research protocol was approved by the ethics committees of Aminu Kano Teaching Hospital, Kano State Hospitals Management Board and Cardia Heart Clinic, Kano, Nigeria. The study conformed to the ethical guidelines of the Declaration of Helsinki, on the principles for medical research involving human subjects [24]. Inclusion criteria for the patients were: (i) new diagnosis of PPCM before commencement of medical treatment; (ii) presenting within nine months *postpartum*; (iii) at least 18 years of age; and (iv) giving written informed consent. To be included, the controls had to satisfy the following criteria: (i) being apparently healthy; (ii) no past history of any cardiac disease or systemic hypertension (except if pregnancy-induced during the last pregnancy); (iii) having normal electrocardiogram (ECG); (iv) presenting to the study centers within nine months *postpartum* for routine immunizations for their children; and (v) giving written informed consent. Subjects were excluded if their results for serum selenium and ceruloplasmin were not available. PPCM was defined according to the recommendations of the HF Association of the European Society of Cardiology Working Group on PPCM, but LV systolic dysfunction as LVEF <50% [3].

At the study sites, physicians and obstetricians were approached and requested to refer all patients with suspected PPCM to the principal investigator (PI) for further evaluation at no cost. Patients were then interviewed, clinically evaluated and recruited consecutively. Hospital in-patients with PPCM were clinically evaluated and underwent investigations within the first 48 h of admission. Demographic data, relevant aspects of history and physical signs, results of investigations, medications, co-morbid conditions, and complications were included in a detailed questionnaire. For each subject, a 12-lead electrocardiogram (ECG) at rest and trans-thoracic echocardiogram (for PPCM patients only) were carried out by the PI at the study centers according to standard recommendations [3]. The echocardiographic examination was carried out using Sonoscape S8 Doppler Ultrasound Machine (Shenzhen, China, 2010) [25,26]. In view of the lack of facilities in Nigeria, serum samples for selenium and ceruloplasmin assays were frozen and sent to Metropolis Diagnostic Centre, Metropolis Healthcare Ltd., New Delhi, India. Serum selenium was measured using the Inductive Coupled Plasma Mass Spectrometry Method, while ceruloplasmin was measured using the Nephelometry Method with BN Prospec Machine (Made by Siemens).

Patients were followed up by the physicians in the respective study centers for six months, and the PI re-evaluated the patients at the completion of the six months including ECG and echocardiogram examinations, but blood tests were not repeated. A high attrition rate of at least 40% was anticipated given that many of the patients did not have contact telephone or might not have money to pay for transportation or because of the frequent terrorist attacks on Kano City, including the main study center (MMSH).

The reference range of serum selenium and ceruloplasmin at the Metropolis Laboratory were 74.0–90.0 µg/L and 20.0–60.0 mg/dL, respectively. In this study, low serum selenium was defined as values <70 µg/L, which seems to be the minimal selenium level that is critical for expression of key antioxidant enzymes such as glutathione peroxidases and the thioredoxin reductases [27,28]. Increased ceruloplasmin level was defined as >60 mg/dL.

Anemia was defined as hemoglobin <12 g/dL, according to the recommendation of World Health Organization in non-pregnant women [29].

### 3.2. Statistical Analysis

Frequencies, mean, median and inter-quartile ranges were used to describe patients’ characteristics. Chi-squared, Fisher’s exact, Student *t*- and Mann-Whitney *U* tests were used to compare categorical and continuous variables as appropriate. Skewness of continuous variables was corrected using Log_10_ transformation. Binary logistic regression models were used to assess for association between low selenium and variables of interest, and values were expressed as Odds Ratios (OR) and 95% Confidence Intervals (CI). The statistical analysis was carried out using SPSS version 16.0 software (Chicago, IL, USA). A *p*-value <0.05 was considered as minimum level of statistical significance.

## 4. Conclusions

The present study has shown that low serum selenium and rural residency are highly significant risk factors for PPCM in Kano, north-western Nigeria. Rural residency increases the odds of having critically low serum selenium levels by up to 2.77-fold. The study has also shown that ceruloplasmin, socio-economic indices, multiparity, pregnancy-induced hypertension, obesity and twin pregnancy are not risk factors for PPCM in the study area. In addition, traditional birth practices that have previously been associated with PPCF are not PPCM risk factors. Baseline serum levels of selenium and ceruloplasmin are not associated with six-month mortality. Future community comparative studies are urgently needed to further explore the relationship between selenium deficiency and PPCM, in view of its high prevalence in sub-Saharan Africa and associated morbidity and mortality.
